# Chemical and Biological Characteristics of *Oxytropis pseudoglandulosa* Plant of Mongolian Origin

**DOI:** 10.3390/molecules26247573

**Published:** 2021-12-14

**Authors:** Tuya Narangerel, Radosław Bonikowski, Konrad Jastrząbek, Alina Kunicka-Styczyńska, Aleksandra Plucińska, Krzysztof Śmigielski, Iwona Majak, Adrian Bartos, Joanna Leszczyńska

**Affiliations:** 1 Institute of Natural Products and Cosmetics, Lodz University of Technology, Stefanowskiego 2/22, 90-537 Lodz, Poland; tuya.narangerel@p.lodz.pl (T.N.); radoslaw.bonikowski@p.lodz.pl (R.B.); konrad.jastrzabek@p.lodz.pl (K.J.); 2 Institute of Fermentation Technology and Microbiology, Lodz University of Technology, Wolczanska 171/173, 90-924 Lodz, Poland; alina.kunicka@p.lodz.pl (A.K.-S.); aleksandra.plucinska@dokt.p.lodz.pl (A.P.); 3 Department of Environmental Biotechnology, Lodz University of Technology, Wolczanska 171/173, 90-924 Lodz, Poland; krzysztof.smigielski@p.lodz.pl; 4 Institute of Food Technology and Analysis, Lodz University of Technology, Stefanowskiego 2/22, 90-537 Lodz, Poland; iwona.majak@p.lodz.pl; 5 Department of Bioinorganic Chemistry, Medical University of Lodz, Muszynskiego 1, 90-151 Lodz, Poland; adrian.bartos@umed.lodz.pl

**Keywords:** *Oxytropis pseudoglandulosa*, medicinal plant, essential oils, volatile oils, lipids, antimicrobial, Mongolia

## Abstract

*Oxytropis pseudoglandulosa* is used in Mongolian traditional medicine due to its numerous reported health-promoting effects. To date, there are very few scientific reports that describe this species. In this article, its volatile oil composition, lipid extract composition, total phenolic and flavonoid content, antibacterial and allergenic properties are elucidated for the first time. Hexadecanoic acid, fokienol and tricosane were determined as the most notable components of the volatile oil, at 13.13, 11.46 and 5.55%, respectively. Methyl benzoate was shown to be the most abundant component of lipid extract at 40.69, followed by (E)-prop-2-enoic acid, 3-phenyl- and benzenepropanoic acid, at 18.55 and 9.97%. With a TPC of 6.620 mg GAE g^−1^ and TFC of 10.316 mg QE g^−1^, the plant extract of *O. pseudoglandulosa* indicated good antioxidant activity measured by IC_50_ at 18.761 µg mL^−1^. Of the 12 tested microorganisms, *B. subtilis* and *S. cerevisiae* were the shown to be most susceptible to the plant extract, with MIC at 2.081 and 0.260% (*v*/*v*), respectively. Bet v 1—a major birch pollen allergen found in plant-based foods—was determined to be at 192.02 ng g^−1^ with ELISA. Such a wide spectrum of biological activity indicated by *O. pseudoglandulosa* lends credence for its application in food industry. Its exerted antioxidant and antimicrobial effects could improve preservation of low-processed food dedicated for consumers afflicted with allergies. Hexadecanoic acid supplemented in foods with dietary plant extracts could add to the potential anti-inflammatory impact. The analysis of lipid makeup suggests *O. pseudoglandulosa* extract could also be considered as natural pesticide in organic farming.

## 1. Introduction

The *Oxytropis* genus consists of over 300 species, nearly half of which are considered native to the territory of China [1]. More than 90% of these are found in the Siberian forest taiga with many of them being endemic species. Owing to their high adaptability, they can be located in diverse types of environment marked by continental climate, such as grasslands, woodlands, prairies, and swamps [2].

Many of the *Oxytropis* plants are used in traditional medicine as a form of treatment for a variety of diseases and their symptoms, including inflammation, cold, swelling and bleeding. Sources published a decade ago report over 127 chemical components isolated from genus *Oxytropis*, including i.a. flavonoids, flavonones, chalcones, isoflavones, isoflavanones, dihydroflavones, alkaloids, saponins, or lignans, all of which can be attributed biological and pharmacological activities [3].

In this study we report on the chemical composition of volatile oil and lipid extracts of *O. pseudoglandulosa* plant species as well as its total phenolic and flavonoid content, antimicrobial properties and allergenicity. Despite the long history of this natural remedy, this is the first time scientific data is published on the chemical compounds underlying its medicinal application.

## 2. Results

### 2.1. Spectrophotometric Determination of Total Phenolic and Flavonoid Content

Phenols and polyphenols are bioactive plant compounds with an aromatic ring bearing one or multiple hydroxyl groups in their structure. They occur naturally or in the form of synthetic bioactive analogues. Total phenolic content was determined in *O. pseudoglandulosa* at 6.620 mg GAE g^−^1 (milligrams of gallic acid equivalents per gram of dry plant mass). Flavonoids are considered the largest group of naturally occurring phenolic compounds. Total flavonoid content in *O. pseudoglandulosa* was found to be 10.316 mg QE g^−1^ (milligrams of quercetin equivalents per gram of plant dry mass).

### 2.2. Antioxidant Activity

Strong antioxidant activity is associated with a high amount of total phenolic content. The IC_50_ (the concentration at which free radical scavenging activity corresponds to 50%) is a measure of antioxidant potential. It was determined with the use of DPPH method. The output IC_50_ equaled 18.761 µg mL^−1^ in *O. pseudoglandulosa* extract. Trolox— a standard antioxidant—yielded a comparable IC_50_ value at 19.37 µg mL^−1^.

### 2.3. GC-MS Determination of Volatile Oil Composition

**Figure 1:**
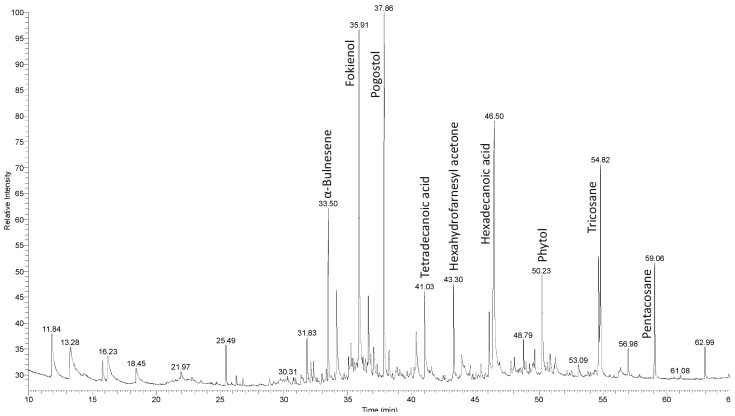
GC-MS chromatogram of an essential oil of *O. pseudoglandulosa*.

Twenty seven chemical components of volatile oil were identified in *O. pseudoglandulosa* using GC-MS analysis. It accounts for 82.77% total volatile oil composition. As shown in Table 1, the most notable compounds included hexadecanoic acid (13.13%), followed by fokienol (11.46%), pogostol (9.85%), tricosane (5.55%), elemicin (4.40%), prasterone (4.33%) and α-bulnesene (4.31%). These can be identified on chromatograms shown in Figure 1.

### 2.4. GC-MS Determination of Lipids

**Figure 2:**
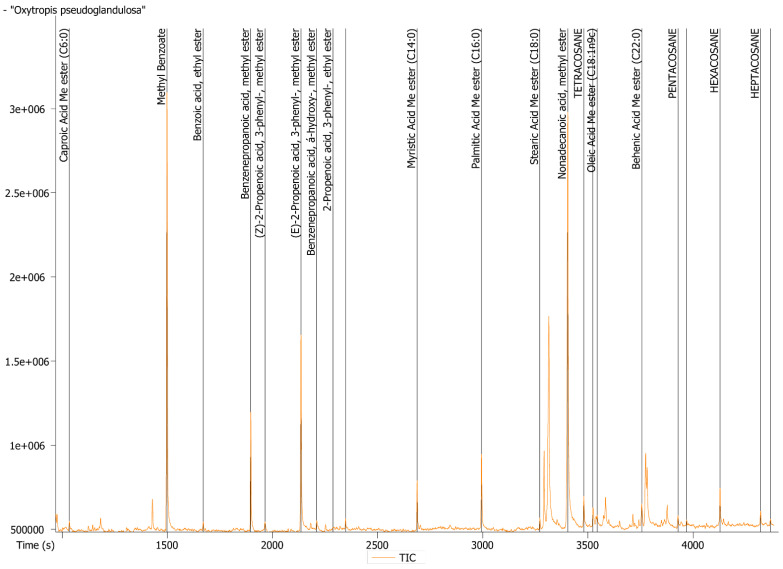
GC/MS analysis of *O. pseudoglandulosa* lipid composition.

Twenty one lipid compounds were identified. As listed in Table 2, fatty acids, (18.74%), phenylpropanoid (C6-C3) and its derivatives (31.14%) as well as acyclic alkanes (7.34%) were determined as the three major groups of compounds in *O. pseudoglandulosa*. Methyl benzoate (C6-C2) (40.69%) was the most abundant compound in the plant sample, followed by trans-2-propenoic acid,3-phenyl (18.55%), benzenepropanoic acid (9.97%), palmitic acid (6.77%), myristic acid (4.02%) and hexacosane (3.75%). A small amount of monounsaturated fatty acids (oleic acid) was determined in the oil extract. Detailed lipid identification data was shown in chromatogram in Figure 2.

### 2.5. Antimicrobial Properties

Ethanol plant extract was evaluated for antimicrobial activity against four strains of Gram-positive bacteria (*Bacillus subtilis* ATCC 6633, *Staphylococcus epidermidis* ATCC 12228, *Staphylococcus aureus* ATCC 6538 and *Micrococcus flavus* LOCK 0849) and four strains of Gram-negative bacteria (*Pseudomonas aeruginosa* ATCC 45442, *Pseudomonas fluorescens* PCM 2123, *Escherichia coli* ATCC 8739 and *Enterobacter aerogenes* ATCC 13048) as well as four strains of fungi (*Candida vini* LOCK 0008, *Saccharomyces cerevisiae* LOCK 0119, *Aspergillus niger* LOCK 0440 and *Penicillium expansum* LOCK 0535). The result of the study was expressed as minimum inhibitory concentration (MIC) and presented in Table 3.

The evaluation of antimicrobial activity in *O. pseudoglandulosa* revealed that the inhibitory concentration of plant extract against Gram-positive bacteria was lower relative to Gram-negative bacteria. Gram-positive *B. subtilis* (2.081%) and Gram-negative *P. fluorescens* (4.162%) were shown to be the most susceptible, followed by *S. epidermidis*, *S. aureus*, *M. flavus* (4.162%) as well as *P. aeruginosa*, *E. coli*, *E. aerogenes* (8.325%). *S. cerevisiae* yeasts were reported as the most susceptible of the tested fungal strains.

### 2.6. Ingestive Allergenicitya

Bet v 1 and profilin are major birch pollen allergens that commonly occur in plant-based foods. Determination performed with ELISA method indicated the content of Bet v 1 in *O. pseudoglandulosa* at 192.02 ng g^−1^, while the content of profilin indicated values below detection limit.

## 3. Discussion

In recent years, the traditional remedies exhibit peaking popularity due to their possible commercial application for medicinal or healthcare purposes [4]. This seems to be especially true for the natural resources of oriental heritage, where products are marked by the long history of their use and little to no scientific foundation performed on their bioactive properties [5,6,7]. *Oxytropis* is a heavily understudied plant genus with very little attention drawn to it from researchers. To set our results into the context of other reports, it was necessary to refer to the articles far past the timeframe of the last decade. Poorly explored as it is, however, the researched plant holds a lot of potential for scientific investigation and discovery.

High levels of phenolic and flavonoid compounds indicate potentially antioxidant and anticancer properties of a medicinal plant [8]. With a TPC of 6.620 mg GAE g^−1^ and TFC of 10.316 mg QE g^−1^, the plant extract of *O. pseudoglandulosa* exhibits exceptionally good antioxidant properties, as concluded from the corresponding IC_50_ at 18.761 µg mL^−1^. These can be confronted with research on *Oxytropis falcate* Bunge by Jiang et al., which seems to be far less potent radical scavenger. Authors indicated their most efficient radical scavenging IC_50_ at as much as 2050 µg mL^−1^ when plant was extracted with ethyl acetate [9]. For a comparison, IC_50_ determined across 11 species of *Thymus* genus Mongolian medical plants indicated values in the range of 273.8 to 679.3 µg mL^−1^ and a maximum TPC and TFC at 37.62 mg GAE g^−1^ and 8.70 mg QE g^−1^, respectively [10]. Rather poor TFC and high antioxidant potential in *O. pseudoglandulosa* could therefore be an indication of a plant being a significantly more capable free radical scavenger due to its highly bioactive profile rather than the quantity of phenolics in its composition. In another study, Mongolian scientists researched on 52 medicinal plants with DPPH assay and found *Saxifraga spinulosa* to be the most promising antioxidant with IC_50_ at 14.98 µg mL^−1^, which is comparable with our findings on *O. pseudoglandulosa* [11]. Also in this case, TPC and TFC values were visibly higher (121.42 ± 14.33 mg GAE g^−1^ and 37.27 ± 3.05 mg RE (rutin equivalents) g^−1^, respectively), suggesting particularly strong antioxidant effect in our tested plant species despite lower TPC.

In our work, the composition of the essential oil *Oxytropis pseduglandulosa* has been tested for the first time. The makeup of volatile oil in *O. pseudoglandulosa* encompassed hexadecanoic acid, fokienol and tricosane as the most notable components, at 13.13, 11.46 and 5.55%, respectively. Chemical composition of a volatile oil is dependent on a large number of variables, including the particular species, specific sample parts (leaves, flowers, fruit and root), geographical location, cultivation environment, drying methods (sun, shade and oven), distillation process (solvent type, extraction time), distillation methods (hydro, water and steam) plant age, time of harvesting (beginning and end of flowering and fruiting) [12,13,14,15]. This is why a direct comparison between two plant species may not be conclusive. Since there are so few reports on *Oxytropis* in general, it is worth to note the outcome of other cases of research on this genus. *Oxytropis kuchanensis* volatile oil was shown to contain limonene and caryophyllene oxide as the most abundant ingredients at 11.4 and 9.6%, respectively. No shared compounds were found when comparing our data with the quoted report [15]. In *Oxytropis falcate* Bunge, on the other hand, the most notable volatile oil components included viridiflorol (11.5%), (E)-nerolidol (8.2%), ethyl hexadecanoate (6.5%), tricosane (5.6%) and spathulenol (5.4%) [16]. That plant has drawn a lot of focus recently due to its reported pro-apoptotic and anti-proliferative properties towards human hepatic cancer cells [17].

In our research, hexadecanoic acid was a dominant phytocompound in the extracted volatile oil. In Indian traditional medicine it is known to hamper the release of inflammatory mediators such as prostaglandin E2, IL-6, IL-1β, TNFα, and nitric oxide and to prevent a number of conditions, such as rheumatoid arthritis, bronchial asthma, ulcerative colitis, systemic lupus erythematosus, psoriasis, and Crohn’s disease [18]. Fokienol was reported to constitute over 20% of *Dittrichia viscosa* L. volatile oil, the effects of which are antiviral, antioxidant, anti-inflammatory and gastric antiulcerous [19]. It was also found to be the most dominant component of *Centaurea damascena*, the volatile oil of which was suggested as an advantageous adjuvant in the antibiotic treatment of pneumonia [20]. The same antibacterial properties can be attributed to tricosane [21].

The lipid composition of Oxytropis pseduglandulosa was tested for the first time. Lipid extract of *O. pseudoglandulosa* indicated over 40% methyl benzoate contribution. From what is known about the compound, it serves as an active green pesticide against several invasive species, such as *Halyomorpha halys* or *Drosophila suzukii* [22] and as a cuing molecule in plant communication [23]. It was reported as one of the main ingredients of *Cananga odorata* oil, a food additive with a demonstrated antibacterial and antioxidant properties [24]. Although the main component is approved for ingestion [24], the co-occurrence of saturated fatty acids in the lipid extract of *O. pseudoglandulosa* hinders its safe oral administration [25].

Ethanol extract of *O. pseudoglandulosa* exhibits antimicrobial properties in a mostly consistent fashion. Three out of four tested Gram-positive bacteria showed inhibited growth due to exposure to phytochemicals at the extract concentration of 4.162%. Twice the amount was sufficient to inhibit the growth of three out of four tested Gram-negative bacteria. Yeasts *S. cerevisiae* and *Candida* sp. displayed the most susceptibility of all tested microorganisms. Similar outcome was reported in the case of *Oxytropis falcate* Bunge, where antibacterial impact was mostly attributed to the content of rhamnocitrin, kaempferol, rhamnetin and 2′,4′-dihydroxychalcon [26] as well as in the case of *Oxytropis* *myriophylla* [27]. Later it was further corroborated that secondary metabolites of the species, including the most bioactive compounds—91 flavonoids isolated to date [27,28] as well as alkaloids inherent to the plant composition [29], are responsible for antibacterial properties among other reported activities, such as antioxidant, anti-tumor, anti-cardiovascular disease, and hemostatic.

Birch trees produce large amounts of pollen. Birch pollen allergy incidence in European population is estimated at up to 22.4%. Its eliciting mechanism is dominated by a single allergen known as Bet v 1. As much as 70% patients diagnosed with birch pollen allergy also react to at least one Bet v 1—associated allergenic food source, such as fruits, vegetables or nuts [30,31]. It is thus a common health threat. Bet v 1 was determined in *O. pseudoglandulosa* at 192.02 ng g^−1^. In a related report by Aninowski et al., we reported the content of Bet v 1 allergen protein determined in cumin, fennel, parsley, anise and coriander at 520–1540, 500–1400, 630–980, 550–1150 and 600–860 ng g^−1^, respectively [32]. In this context, *O. pseudoglandulosa* can be considered as a much less of a threat to those afflicted with birch pollen allergy than what was reported in the case of herbal spices of regular culinary use. Its safe oral administration must be, however, tested to ensure the accepted intake threshold.

In this work, the chemical composition of *O. pseudoglandulosa* was tested for the first time. Antioxidant and antimicrobial properties demonstrated by *O. pseudoglandulosa* lay the ground for the potential application of the plant extract as a supplementary agent in manufacturing of low-processed food preservation. Inclusion of a natural bioactive additive could allow to limit the use of less tolerable substances thought to extend the longevity of food products. This becomes even more significant given the low allergenic potential of *O. pseudoglandulosa*. Apart from that, the occurrence of hexadecanoic acid could point to a possible anti-inflammatory effect, which further expands the scope of health-promoting advantages of the plant. *O. pseudoglandulosa* lipid extracts may also be considered as natural pesticide in organic farming. To our knowledge, this is the first report presenting a wide spectrum of biological activity of *O. pseudoglandulosa* molecular components. Further research will be undertaken to focus on a potential pharmacological application of the plant as a functional food additive.

## 4. Materials and Methods

### 4.1. Sample Collection

Medicinal plant *O. Pseudoglandulosa* was obtained from the local market in Ulaanbaatar, Mongolia. Collection took place in Ulaan-Uul sum, Khuvsgul province in August 2016. Botanical identification of the plant was confirmed by Dr. Urgamal Magsar from the Institute of General and Experimental Biology (Mongolian Academy of Sciences). Plant material was subjected to drying in the shade at 15–17 °C for three weeks. The aerial part of the plant was gently ground using a pestle and mortar and packed in PE plastic ziplock bags. It was stored in a dark place at room temperature at all times.

### 4.2. Extraction of Phenolic and Flavonoid Compounds

Weighted 0.500 g portions of finely ground samples were extracted with 12 mL of 50% methanol (*v*/*v*). Methanol was prior acidified with 1% of formic acid (*v*/*v*) and mixed using a shaker (TTS 2, Yellow Line, IKA-Werke, Staufen, Germany) at room temperature for one hour. Acidification was performed to avoid oxidation of polyphenols. The supernatant was then separated via centrifugation at 10,000 rpm, for 5 min (MPW-251, Med instrument, Warsaw, Poland) and passed through paper filters (BOECO Germany Grade 3 hw). The sediment was re-extracted with 10 mL of 50% methanol (*v*/*v*) and separation process was repeated. Finally, collected supernatants were combined into a 25-mL volumetric flask and filled up to the mark with 50% methanol. They were kept in the freezer (−20 °C) until tested.

### 4.3. Total Phenolic and Flavonoid Content

To determine total phenolic content, 50 µL of the 50% methanol extract was mixed with 250 µL of Foline–Ciocalteu’s reagent and 2.5 mL of 20% sodium carbonate solution in a 25 mL volumetric flask and filled with deionized water up to the graduation mark. The mixture was incubated for one hour in a dark place. The absorbance of the mixture was measured at 720 nm using a spectrophotometer HP8453 (Hewlett Packard/Agilent, Houston, TX, USA). All samples were tested in triplicates. The results were expressed in milligrams of gallic acid per gram of plant dry mass.

Gallic acid 250 µg mL^−1^ standard solution was used to prepare reference dilutions at a concentration range from 5 to 150 µg mL^−1^. The total phenolic content of the samples was estimated using calibration curves pre-determined with gallic acid.

For total flavonoid content determination, 500 µL sample extract was mixed with 1.5 mL of 80% methanol (*v*/*v*), 100 µL of 10% AlCl_3_, 100 µL of CH_3_COONa (1 M) and 3.0 mL deionized water. The incubation was performed in the dark place for 30 min. Absorbance was measured at 415 nm using a spectrophotometer HP8453 (Hewlett Packard/Agilent, USA). All samples were tested in triplicates. The results were expressed in milligrams of quercetin equivalents (QE) per gram of dry plant mass.

For the preparation of calibration curve, 200 µg mL^−1^ of quercetin was dissolved in 80% methanol (*v*/*v*) and used as stock solution. Dilutions were prepared at concentrations between 5 and 100 µg mL^−1^. Total flavonoid content was estimated using calibration curve pre-determined with quercetin.

### 4.4. Free Radical Scavenging Activity and Calculation of the IC_50_

First, 0.1 mM of DPPH solution was prepared through dilution in 80% methanol (*v*/*v*). Trolox at a concentration of 80 µg mL^−1^ was used as a standard antioxidant. The DPPH solution at the volume of 2.0 mL was combined with 2.0 mL of the plant extract. The same was applicable for standard solution, prior adjusted to the following concentrations: 5, 10, 20, 30, 40 and 50 µg mL^−1^. The mixture was blended on vortex and allowed to stand in the dark for 30 min. Absorbance was measured at 517 nm using a UV–VIS spectrophotometer HP8453 (Hewlett Packard/Agilent, USA). In the blank control, the sample was substituted with deionized water. All analyses were performed in triplicates. The DPPH radical scavenging activity was calculated from the following equation:

RSC, % = 100 (A_0_ − A)/A_0_ (1)

where: A—average absorbance of the sample

A_0_—average absorbance of a control (DPPH)

**Figure 3:**
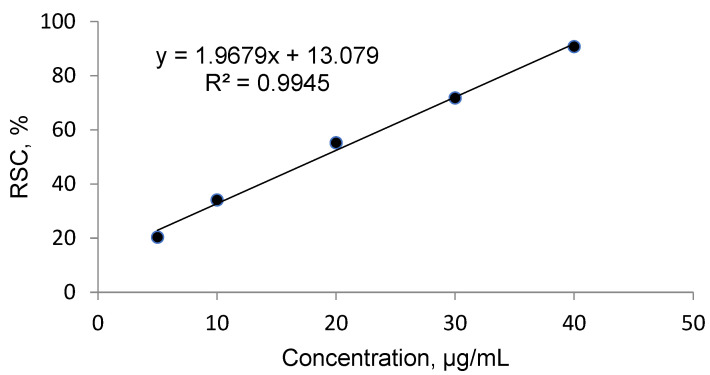
Radical scavenging activity determined in *O. pseudoglandulosa* extract.

**Figure 4:**
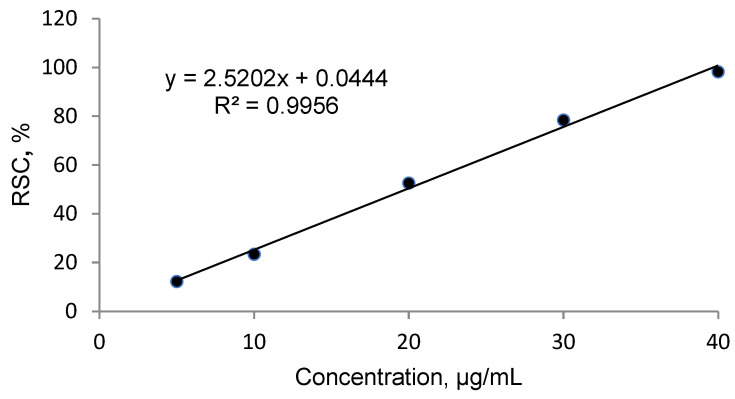
Radical scavenging activity determined in Trolox antioxidant standard solution.

Radical scavenging capabilities of *O. pseudoglandulosa* extract and Trolox antioxidant standard solution were shown in Figure 3 and Figure 4, respectively.

The inhibitory concentration was plotted against the free radical scavenging activity measured in the samples. The radical scavenging activity of the methanol extract samples was calculated using the linear (y = ax + b) equation plotted with the DPPH values, with Y value being 50 and the X point being IC50 value. The value was expressed in micrograms of Trolox equivalents per mL of methanol extract.

### 4.5. GC-MS Determination of Volatile oil Components

Essential oils were isolated through hydro-distillation from 5.00 g dry plant weighted portions using a Clevenger-type apparatus. The makeup of volatile compounds in the oil composition was determined using Thermo Trace GC Ultra/DSQ II (Thermo Fisher Scientific, Waltham, MA, USA). Operating parameters of the gas chromatography were set up as follows: column—non-polar stationary phase Rxi–1 ms (length 60 m, internal diameter 0.25 mm, film thickness 0.25 μm, Restek Corp., Bellefonte, PA, USA), injector temperature: 280 °C, FID detector temperature: 300 °C, carrier gas—helium, constant pressure 300 kPa and split ratio 1:100, oven temperature program was 50 to 300 °C at 4°/min. Mass spectrometry parameters: ion source temperature 200 °C, ionization energy 70 eV. The quantity of the individual components was expressed as a percentage of the essential oil and was achieved using flame-ionization detector connected through the MS-FID splitter (SGE Analytical Science, Ringwood, Melbourne, VIC, Australia). Databases from the NIST Library, Wiley 8th edition and the Adams 4th edition were used. All samples were tested in triplicates.

### 4.6. Extraction of Lipids

To obtain lipid extracts, weighted portions of 15 g dry plant were subjected to extraction with isooctane. Afterwards, the solvent was slowly vaporized using a rotary vacuum evaporator. The residue (lipid extract) from the distillation flask was collected into a glass test tube. The extract was stored at 4 °C.

### 4.7. GC-MS Determination of Lipids

The portion of 10.0 mg of the extracted sample was mixed with: 100 µL of methyl nonadecanoate solution in MTBE and 100 µL of TMSH. The solution was incubated at 80 °C for 30 min and 1 µL of the output mixture was subjected to GC-MS analysis. The GC-MS determination was performed with a liquid chromatograph coupled with a time-of-flight mass spectrometry detector (Pegasus 4D, LECO, St. Joseph, MI, USA).

Operating parameters were set up as follows: Stabilwax capillary column (fused silica) 20 m × 0.18 mm diameter, 0.18 µm film thickness, gradient temperature: 50–245°C at 4° min^−1^, carrier gas—helium and flow rate: 1 mL min^−1^. Identification of lipids was based on the comparison of their mass spectra with data available from commercial database (37 Component FAME Mix, Supelco, Saint Louis, MO, USA. Cat. No. CRM47885). The content of the individual components was expressed as a percentage of total extracted lipids. All samples were tested in triplicates.

### 4.8. Antimicrobial Activity

Weighted portion of 0.25 g dry plant was extracted with 1.00 mL of 95% ethanol (*v*/*v*) using a shaker (TTS 2, Yellow Line, IKA-Werke, Staufen, Germany) at the room temperature for 24 h. The supernatant was passed through filter paper (BOECO Germany Grade 3 hw). Ethanol was evaporated gradually and the residue was collected.

The following microorganisms were used in the study: bacteria: *Bacillus subtilis* ATCC 6633, *Staphylococcus epidermidis* ATCC 12228, *Staphylococcus aureus* ATCC 6538, *Micrococcus flavus* LOCK 0849, *Pseudomonas aeruginosa* ATCC 45442, *Pseudomonas fluorescens* PCM 2123, *Escherichia coli* ATCC 8739, *Enterobacter aerogenes* ATCC 13048 as well as fungi: *Candida vini* LOCK 0008, *Saccharomyces cerevisiae* LOCK 0119, *Aspergillus niger* LOCK 0440, *Penicillium expansum* LOCK 0535. The strains originated from American Type Culture Collection (ATCC), Polish Collection of Microorganisms Institute of Immunology and Experimental Therapy Polish Academy of Sciences (PCM) and Collection of Pure Cultures of the Institute of Fermentation Technology and Microbiology Technical University of Lodz ŁOCK 105 (LOCK). The microorganisms were activated through double passaging: bacteria on trypticase soy agar medium (TSA for 37 °C, 48 h; Oxoid, Basingstoke, UK), yeast and molds on Sabouraud dextrose agar medium (SDA for 28 °C, 72 h yeast and 7 days molds; bioMerieux, Warsaw, Poland).

The test for antimicrobial activity of extracts was conducted with microdilution method according to CLSI recommendations (CLSI, 2015) [33] using 96-wells microtiter plates. The volume of 100 μL of pure extracts were diluted with 100 μL of TSB medium (Trypticase Soy Broth, Merck, Germany) and then 100 μL of microorganism suspension was added. Previously prepared suspensions of the tested bacteria and yeast were used in a physiological salt solution (0.85% NaCl), while the molds were used in a physiological salt solution with the addition of 0.5 g L^−1^ polysorbate 80 R and standardized to the density of 10^6^ CFU mL^−1^. Next, 2-fold subsequent dilutions of the extract were prepared. The extract concentrations tested were in the range of 33.333–0.130% (*v*/*v*). The following negative controls were conducted: pure extracts without microorganisms and the culture with suspension of microorganism only. As positive controls, bacteria and fungi cultures were used with the addition of novobiocin (0.5 μg mL^−1^) and cycloheximide (0.2 μg mL^−1^), respectively.

All plates were incubated in 30 °C for 24 h or in 28 °C for 48 h, in the case of bacteria and fungi, respectively. After incubation, the results of experiment were examined with the use of 0.01% solution of resazurin. The volume of 10 μL solution was added to each well on the plate and then incubated for 2 h in 30 °C. The viability of microorganisms was estimated macroscopically, assuming that red color of the well indicates presence of living organisms, while blue color stands for a lack of the living microorganism. On the bases of color changes the MIC values of extracts were determined as the lowest extract concentration inhibiting the growth of microorganisms. The experiments were conducted in triplicates for each microorganism tested.

### 4.9. Extraction of Food Allergens

Weighted portions of 0.100 g plant material were ground against and pressed through a filter 60 times using a plastic rod with twisting force. Then, 1000 µL of the native lysis buffer was added and the sample was ground 60 times more with twisting force. The filter cartridge was transferred to the refrigerator for 12 h. Afterwards, the supernatant was subjected to centrifugation at 10,000 rpm (MPW-251, Med instrument, Warsaw, Poland) for 3 min and deposited in the freezer (−20 °C).

### 4.10. Allergenic Protein Content Determination

Allergenicity of the extracts was determined with ELISA method. The volume of 100 μL supernatant was used to coat 96-well microplate (SPL Lifesciences, South Korea) and was left for an overnight incubation at 4 °C. On the next day, the microplate was washed with 3 × 350 μL PBS–T washing buffer (pH = 7.4 PBS, 0.1% Tween-20). Afterwards, 3% low fat milk in PBS–T washing buffer was used to block unbound sites in the wells through 2-h incubation at room temperature. The milk solution was removed and washed with 3 × 350 μL of PBS–T washing buffer. The volume of 100 µL of diluted (1:1000) primary rabbit anti-profilin antibody or diluted (1:500) mouse anti-Bet v 1 antibody was applied and incubated at room temperature for one or two hours, respectively. After incubation, washing cycle was repeated and 100 µL of diluted (1:5000) anti-rabbit secondary antibody or anti-mouse IgG conjugated to alkaline phosphatase was added, followed by one hour incubation at room temperature. The microplate was washed with 3 × 350 μL of PBS–T washing buffer. It was then incubated with pNPP at 100 µL applied per well for one hour at room temperature. The reaction was stopped with 50 µL of 3 M NaOH. Multiscan RC reader (ThermoLabsystem, Helsinki, Finland) was used to measure the absorbance at 405 nm. The allergen protein values were expressed as nanograms per gram of the sample. All samples were tested in triplicates from two extracts.

Calibration curves were plotted for Bet v 1 and profilin. As for Bet v 1, the curve was prepared with standard solutions at concentrations between 0.5 and 50 ng mL^−1^ and was shown to follow equation formula y = 0.0242x − 0.0697 (R^2^ = 0.992). For profilin, the curve was prepared with standard solutions at concentrations range of 0.5 to 100 ng mL^−1^ and it was shown to follow the formula y = 0.0017x + 0.07415 (R^2^ = 0.994). The content of Bet v 1 and profilin allergen proteins in analytical samples were calculated using standard calibration curves.

### 4.11. Statistical Analysis

All obtained results were subjected to statistical analysis using one-way ANOVA analysis, followed by the Dunnett’s test performed using GraphPad Prism 6.0 software (GraphPad Software, Inc., La Jolla, CA, USA) at the significance level of * *p* ≤ 0.05, ** *p* ≤ 0.01, *** *p* ≤ 0.001.

## Figures and Tables

**Table 1:** Chemical components of volatile oil in *O. pseudoglandulosa*.

Compound	Total, %	Compound	Total, %
Oct-1-en-3-one	3.20	(Z)-Hex-3-enyl cinnamate	0.75
Limonene	0.62	Methyl hexadecanoate	0.34
Dec-1-en-3-one	2.05	Sclareoloxide (Cis-B/C)	1.76
Linalool	1.09	Hexadecanoic acid	13.13
Vitispirane	1.01	Kaur-16-ene	1.00
Trans-beta-Ionone	0.69	Methyl linoleate	0.91
α-Bulnesene	4.31	Phytol	3.48
Elemicin	4.40	Prasterone	4.33
Nerolidol	0.82	Tricosane	5.55
Fokienol	11.46	Tetracosane	0.70
Pogostol	9.85	Pentacosane	3.05
Bulnesol	0.77	Hexacosane	0.83
Tetradecanoic acid	2.78	Nonacosane	1.32
Hexahydrofarnesyl acetone	2.57		
		Total identified	82.77

**Table 2:** Identification of lipid compounds in *O. pseudoglandulosa*.

Compound	Total, %	Compound	Total, %
Caproic acid (C6:0)	0.79	Benzenepropanoic acid, α-hydroxy-	1.32
Lauric acid (C12:0)	0.78	(Z)-prop-2-enoic acid, 3-phenyl-	1.19
Myristic acid (C14:0)	4.02	(E)-prop-2-enoic acid, 3-phenyl-	18.55
Palmitic acid (C16:0)	6.77	Propenoic acid, 3-phenyl-, ethyl	0.11
Stearic acid (C18:0)	0.84	Methyl benzoate	40.69
Arachidic acid (C20:0)	2.09	Benzoic acid, ethyl	0.82
Behenic acid (C22:0)	2.46	Tetracosane	1.27
Lignoceric acid (C24:0)	0.45	Pentacosane	1.15
Octacosanoicacid	0.53	Hexacosane	3.75
Oleic acid (C18:1)	1.28	Heptacosane	1.17
Benzenepropanoic acid	9.97		

**Table 3:** Minimal inhibitory concentrations of ethanol extract of *O. pseudoglandulosa* determined with microdilution method.

Gram+	MIC, %(*v*/*v*)	Gram-	MIC, %(*v*/*v*)	Fungi	MIC, %(*v*/*v*)
*M. flavus*	4.162	*P. fluorescens*	4.162	*S. cerevisiae*	0.260
*S. aureus*	4.162	*P. aeruginosa*	8.325	*C. vini*	1.040
*B. subtilis*	2.081	*E. coli*	8.325	*A. niger*	4.162
*S. epidermidis*	4.162	*E. aerogenes*	8.325	*P. expansum*	4.162

## Data Availability

The data presented in this study are available in this manuscript.

## Sample Availability

Samples of the compounds are available from the authors.
